# Knowledge and awareness of nicotine, nicotine replacement therapy, and electronic cigarettes among general practitioners with a special interest in respiratory medicine in China

**DOI:** 10.3389/fmed.2023.1236453

**Published:** 2024-01-08

**Authors:** Qian Zhong, Kang An, Zengxiang Wu, Haijun Zhang, Shengxi Li, Lin Zhang, Caizheng Li, Heting Li, Qi Mei Zhuo Ga, De Ji Yang Zong, Zhenmei An

**Affiliations:** ^1^Department of Endocrinology and Metabolism, West China Hospital, Sichuan University, Chengdu, Sichuan, China; ^2^General Practice Ward/International Medical Center Ward, General Practice Medical Center, National Clinical Research Center for Geriatrics, Multimorbidity Laboratory, West China Hospital, Sichuan University, Chengdu, Sichuan, China; ^3^West China School of Public Health and West China Fourth Hospital, Sichuan University, Chengdu, Sichuan, China; ^4^Department of General Practice, People’s Hospital of Lhasa, Lhasa, Tibet, China

**Keywords:** electronic cigarettes, nicotine, general practitioners with a special interest, knowledge, awareness

## Abstract

**Objectives:**

This study aimed to assess the knowledge and awareness of nicotine, nicotine replacement therapy (NRT), and electronic cigarettes (e-cigarettes) among general practitioners with a special interest (GPwSIs) in respiratory medicine.

**Methods:**

A cross-sectional study was conducted from October 2021 to February 2022. Knowledge and awareness were compared among smokers and non-smokers, as well as different age and gender groups.

**Results:**

The study consisted of 102 GPwSIs from 21 cities in Sichuan Province, China. Most respondents would recommend NRT for long-term use. Only a few believed that e-cigarettes are an effective means of smoking cessation and 71.6% would not recommend e-cigarettes as a substitute for cigarettes to their patients. Additionally, the majority did not regularly provide extensive help to assist patients in quitting smoking and needed smoking cessation counseling training.

**Conclusion:**

GPwSIs in respiratory medicine in China could have a relatively low level of knowledge and awareness regarding nicotine, NRT, and e-cigarettes. The study highlights the need for smoking cessation training among GPwSIs to improve their knowledge and provide better assistance to patients who want to quit smoking.

## Introduction

Tobacco use poses a significant threat to public health worldwide, with smoking causing over 8 million deaths annually, surpassing the combined deaths caused by diseases such as HIV, tuberculosis, and malaria (1). Numerous studies have shown that smoking is strongly linked to chronic diseases such as cardiovascular and respiratory diseases, as well as cancer (2–5). China, a significant contributor to global tobacco production and consumption, is a critical focal point in addressing this health crisis (6). In 2018, the Chinese Center for Disease Control and Prevention conducted a large-scale survey that revealed a smoking prevalence rate of 26.6% among individuals aged 15 and above, which was only slightly lower than the rate of 28.3% reported in 2010 (6). Despite the government’s efforts to promote smoking control policies over the last decade, these figures indicate that a significant number of people in China still engage in smoking (6, 7).

Quitting smoking is considered one of the most effective interventions for promoting health (8). In response to the addictive mechanism of nicotine, various strategies for treating tobacco dependence have been continuously developed and applied, divided into pharmacological interventions and non-pharmacological interventions based on whether drugs are used for withdrawal (9). Nicotine replacement therapy (NRT) is the most widely used pharmacological intervention include nicotine patches, gum, lozenges, inhalers, and nasal sprays (10). The effectiveness of NRT has been validated through multiple clinical trials, which show that NRT can enhance the success rates of smoking cessation. In addition, some drugs are also applied for smoking cessation, such as varenicline, bupropion and cytisine, whose mechanisms of action are similar to NRT (11). Research on e-cigarettes as a tool for reducing nicotine addiction is ongoing, but there is emerging evidence suggesting that they help aid smoking cessation (12, 13). Notably, a study comparing e-cigarettes with NRT found a higher 1-year abstinence rate in the e-cigarette group (18.0%) compared to the nicotine-replacement group (9.9%) (14).

General practitioners (GPs) are of essence in providing appropriate advice and guidance to smokers. The existing evidence indicates that GPs’ knowledge and beliefs about smoking and nicotine may not always be accurate or grounded in scientific evidence (15). Compared to smoking, many GPs overestimate the adverse effects of smokeless tobacco (snus) (16) and NRT (17). For example, a study of GPs in the UK and Sweden found that around 40 per cent of the participants mistakenly believed that nicotine was the primary or second most significant contributor to smoking-related illnesses (18). This misconception can affect the management of smokers, preventing them from using nicotine-based products or other alternatives that are less harmful than smoking.

GPs with a Special Interest (GPwSI) are GPs who have acquired additional training and expertise in a specific clinical area, allowing them to take referrals for specialized cases or provide enhanced services for certain conditions or patient groups (19). The prerequisite for GPwSI to effectively counsel smokers relies on appropriate education and understanding about the impact of smoking on health, as well as all available methods and products that help reduce or quit smoking (20). To our knowledge, there is no study reported from China on this subject. This study aims to assess the knowledge and awareness of GPwSI in respiratory medicine regarding nicotine, NRT, and e-cigarettes.

## Methods

### Population and sample

This cross-sectional study was conducted between October 2021 and February 2022. The population of GPwSI in respiratory medicine is relatively small and obtaining a random sample is difficult in China (21). Therefore, a convenient sampling technique was used to recruit GPwSIs in respiratory medicine for an online survey, through the Western General Practice Network, which includes GPs from primary healthcare settings across Sichuan Province. The sample size was calculated using the online sample size calculator RaoSoft ®. The minimum effective sample for this study was *n* = 97 with a 5% margin error, a confidence interval of 90%, response rate 10% and the total number of general practitioners in Sichuan was 10,394.

Eligible participants for this study were GPs who (1) currently practiced in the registered healthcare facilities at all levels located in Sichuan, China; (2) completed one of the following training programs: a specialty training program in respiratory medicine, academic education in respiratory medicine, or continuing education in respiratory medicine; (3) independently practiced in general practice outpatient clinics; (4) consented to the survey. Excluded practitioners were those who held part-time positions in pharmaceutical companies related to the sales of cessation medications agents.

### Questionnaire design

The questionnaire was designed based on the previous literature (12, 20, 22, 23). In order to enhance the questionnaire’s design and readability, three healthcare professionals were recruited for modification, including two respiratory physicians, a general practitioner. Before the survey, we conducted a small-scale pilot survey, and a total of 20 pilot survey questionnaires were distributed. Feedback from the pilot questionnaire was used to refine the questionnaire for the survey. The researchers created a questionnaire and uploaded it to an online survey tool.^1^ The questionnaire consisted of five main sections, including: (1) demographic and professional characteristics of the participants, such as age, gender, professional title, highest education level, and hospital type; (2) a health risks score for smoking products measured through ‘Unfamiliar’ responses and Likert-type scales, ranging from 1 (low risk) to 5 (high risk); (3) knowledge and attitudes toward nicotine replacement therapy and electronic cigarettes; (4) awareness of the effects of nicotine on smoking-related diseases measured through ‘Unfamiliar’ responses and Likert-type scales, ranging from 1 (low risk) to 5 (high risk); and (5) confidence in providing smoking cessation services, as well as smoking cessation training needs.

With regard to nicotine, the main objective of the questionnaire was to assess its perceived contribution to smoking-related lung cancer and other organ tumors. For NRT, the questions mainly focused on the dosage selection, willingness to recommend long-term use as a smoking substitute, safety compared to tobacco cigarettes and e-cigarettes, and addictiveness compared to e-cigarettes. As e-cigarettes involved novel products, our goal was to assess whether GPwSIs would recommend e-cigarettes as a smoking cessation aid for those who could not or were unwilling to use other methods, as well as evaluate e-cigarettes addictiveness and safety. Finally, participants were asked to report their confidence in their own smoking cessation service capabilities. The survey questionnaire can be found in the Supplementary material (Additional file 1).

### Quality control

To ensure the data credibility, quality control measures were implemented. Participants were only allowed to submit their responses once and were unable to make any changes after submitting. All survey items were required to be completed upon submission. Survey data containing logical errors were checked.

### Ethics statement

This study was approved by the ethical committee, West China Hospital of Sichuan University, Chengdu, China (No. 2021–1745). All participants were informed about the study’s purpose, and they were assured that their data would remain anonymous and confidential.

### Statistical analysis

Descriptive analysis was utilized to characterize the data. Categorical variables were presented as numerical values (%). Differences between categorical variables were assessed using chi-square (*χ*^2^) or Fisher’s exact tests. All statistical tests were two-tailed, and differences were deemed statistically significant when *p* ≤ 0.05. All analyses were performed in SPSS (SPSS version 22, SPSS, Inc., Chicago).

## Results

### Descriptive analysis of the study sample

The descriptive analysis results of the entire study sample are shown in Table 1. A total of 102 GPwSIs in respiratory medicine from 21 cities in Sichuan Province participated in this study. Approximately half were female (49.0%), and the majority aged between 40 and 49 years old (54.9%). The participants had either a bachelor degree (60.8%) or an associate degree (39.2%), with no master or above degree, and most held intermediate professional titles (68.6%). Seventy-eight point 4% of the participants were from tertiary hospitals. Eighteen participants were current smokers, eight past smokers, and seventy-six non-smokers. About half of the GPwSIs reported that they often asked patients about their smoking status, but most did not regularly provide extensive help to assist patients in quitting smoking. Only a small number of participants helped patients quit smoking by means of oral smoking cessation medication (6.9%) or NRT (20.6%). Five point 9% of the participants believed they were fully prepared to provide smoking cessation counseling, while 94.1% believed they needed to receive smoking cessation counseling training.

**Table 1:** Baseline characteristics of all survey participants (*N* = 102).

Participant characteristics		*N*	%
Gender	Male	52	51.0
	Female	50	49.0
Age	20–29	6	5.9
	30–39	29	28.4
	40–49	56	54.9
	50–	11	10.8
Professional title	Primary	12	11.8
	Intermediate	70	68.6
	Senior	20	19.6
Highest education level	Associate	40	39.2
	Bachelor	62	60.8
	Master or above	0	0
Hospital type	Tertiary hospital	80	78.4
	Secondary hospital	19	18.6
	Primary hospital	3	2.9
Smoking status	Non-smoker	76	74.5
	Past smoker	8	7.8
	Current smoker	18	17.6
Have you ever tried to help patients quit smoking? Which of the following methods have you used? (Multiple choice)	Oral smoking cessation medications	7	6.9
	Nicotine replacement therapy (patches, gum, inhalers, nasal sprays, etc.)	21	20.6
	Systematic psychological support	32	31.4
	Other methods	40	39.2
	No attempt to help patients quit smoking	42	41.2
Do you proactively inquire about and record the smoking history of patients in your daily consultations?	Never	4	3.9
	Occasionally	6	5.9
	Sometimes	16	15.7
	Often	53	52.0
	Always	23	22.5

### Beliefs and attitudes toward nicotine, NRT and e-cigarettes

The responses of GPwSIs to nicotine, NRT and e-cigarettes are shown in Table 2. When asked about the smoking cessation drugs approved for use in China, the majority knew about nicotine patch (90.2%) and nicotine gum (92.2%). Regarding the dosage and usage of nicotine patches, only 27.5% of the respondents knew that a higher dose should be used initially and gradually reduced according to the course of treatment. More than half of the respondents believed that long-term (more than 6 months) use of NRT to reduce or quit smoking was safe. Over half of the participants would recommend long-term (more than 6 months) use of NRT to treat those who could not reduce or quit smoking in the short term. The correct dosage of nicotine patches typically starts with the highest dose patch that a smoker can use based on their daily smoking volume. The dosage is then gradually reduced over a period of 8 to 12 weeks, to wean the smoker off nicotine dependence. Significant differences in knowledge regarding the correct dosage and usage of nicotine patches were found between smokers (current and past) and non-smokers (*p* < 0.05) (Supplementary Tables S1).

**Table 2:** Knowledge and awareness of nicotine, NRT, and e-cigarettes (*N* = 102).

Questions	*N*	%
Which of the following smoking cessation medications have been approved for use in China? (Multiple choice)		
Nicotine patch (Right)	92	90.2
Nicotine gum (Right)	94	92.2
Bupropion hydrochloride (Right)	69	67.6
Varenicline (Right)	57	55.9
Which of the following is correct regarding the dosage of nicotine patches?		
Start with a higher dose and gradually decrease it according to the course of treatment. (Right)	28	27.5
Start with a lower dose and gradually increase it according to the course of treatment.	11	10.8
Fixed dose and treatment course.	4	3.9
Do not know	59	57.8
Do you believe that long-term (> 6 months) use of nicotine replacement therapy can reduce smoking or achieve smoking cessation, and is it relatively safe?		
Safe	65	63.7
Unsafe	37	36.3
Do you recommend long-term (> 6 months) use of nicotine replacement therapy to treat those who cannot reduce or quit smoking in the short term?		
Yes	71	69.6
No	31	30.4
Do you think that e-cigarettes are a tool for quitting smoking?		
Yes	30	29.4
No	72	70.6
Do you hold that e-cigarettes can generate addiction?		
Yes	58	56.9
No	9	8.8
Do not know	35	34.3
Would you recommend e-cigarettes as a substitute for cigarettes to smokers?		
Yes	29	28.4
No	73	71.6
Self-evaluation of confidence level in your ability to provide smoking cessation services:		
Completely lacking in confidence	26	25.5
Somewhat lacking in confidence	41	40.2
Somewhat confident	23	22.5
Quite confident	6	5.9
Very confident	6	5.9
Do you need training on smoking cessation?		
Need	96	94.1
No need	6	5.9

Regarding e-cigarette, 29.4% of the respondents believed that they were an effective tool for quitting smoking. More than half (56.9%) agreed that e-cigarettes could lead to addiction, and 34.3% were not aware of the addiction of e-cigarettes. A significant proportion of respondents (71.6%) stated that they would not recommend e-cigarettes as a substitute for cigarettes to their patients. Most lacked confidence in their counseling ability associated with smoking cessation service.

**Figure 1:**
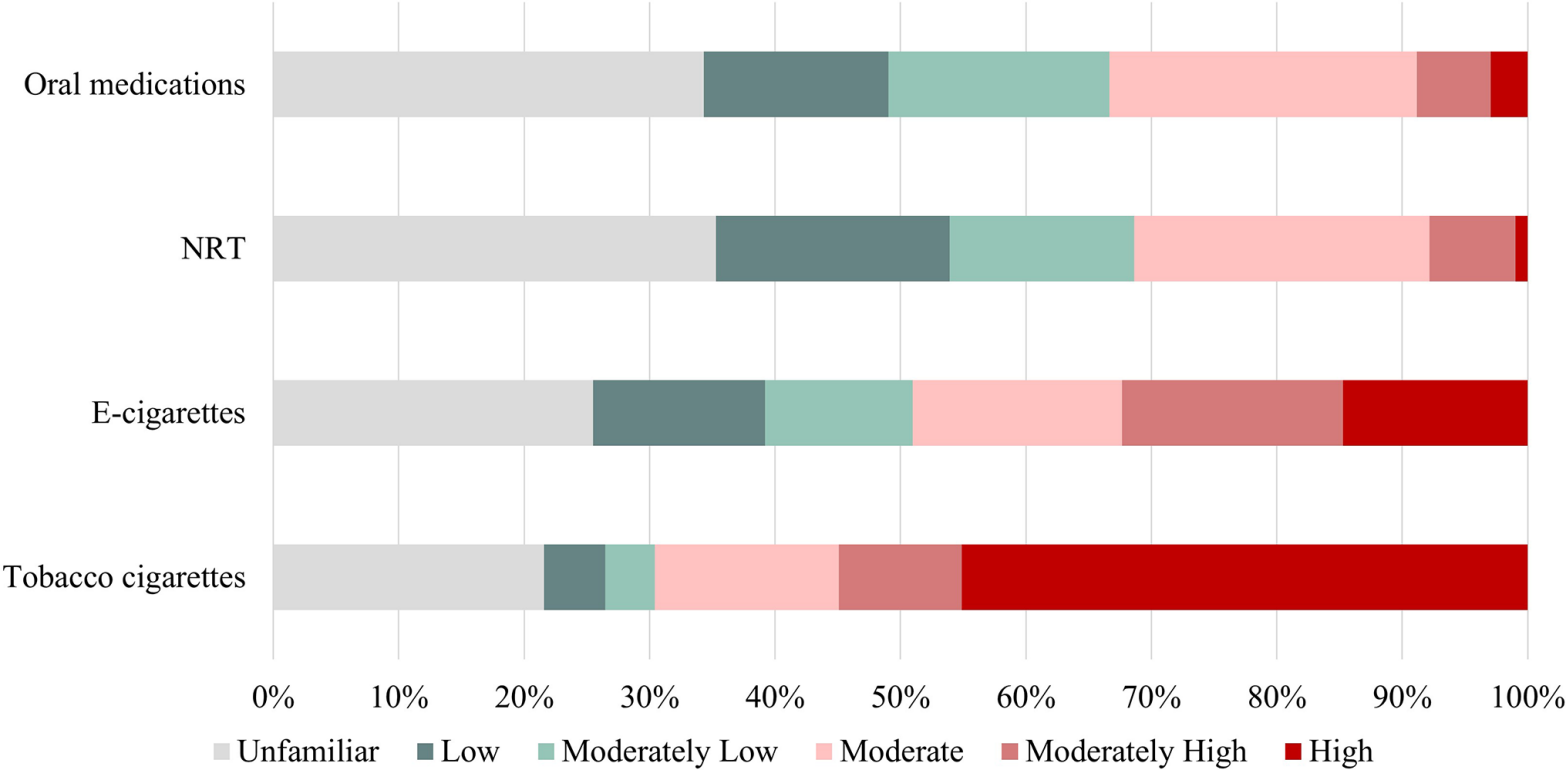
Health risks of smoking products. E-cigarettes, Electronic cigarettes; NRT, Nicotine replacement therapy.

The participants were requested to assess the level of risk associated with tobacco cigarettes and various substitute products, which included e-cigarettes, NRT such as nicotine patches and gum, as well as oral cessation medications like bupropion and varenicline. According to the survey, 54.9% of the respondents considered tobacco cigarettes to pose a moderately high or high health risk, while the figures for e-cigarettes, NRT, and oral cessation medications were 32.4, 7.8, and 8.8%, respectively. On the other hand, 21.6% lack awareness of the health risks associated with cigarettes, while 25.5% are unaware of the harm that e-cigarettes can pose to health. Additionally, 35.3% of people do not have knowledge about the health risks of NRT, and 34.3% are not aware of the potential health risks associated with oral smoking cessation medications. Five individuals believed that smoking had a low health risk. Regarding e-cigarettes, respondents’ ratings were evenly distributed with 13.7, 11.8, 16.7, 17.6, and 14.7% rating them as low, moderately low, moderate, moderately high, and high risk, respectively. Furthermore, 25.5% of participants reported being unfamiliar with e-cigarettes (Figure 1).

**Figure 2:**
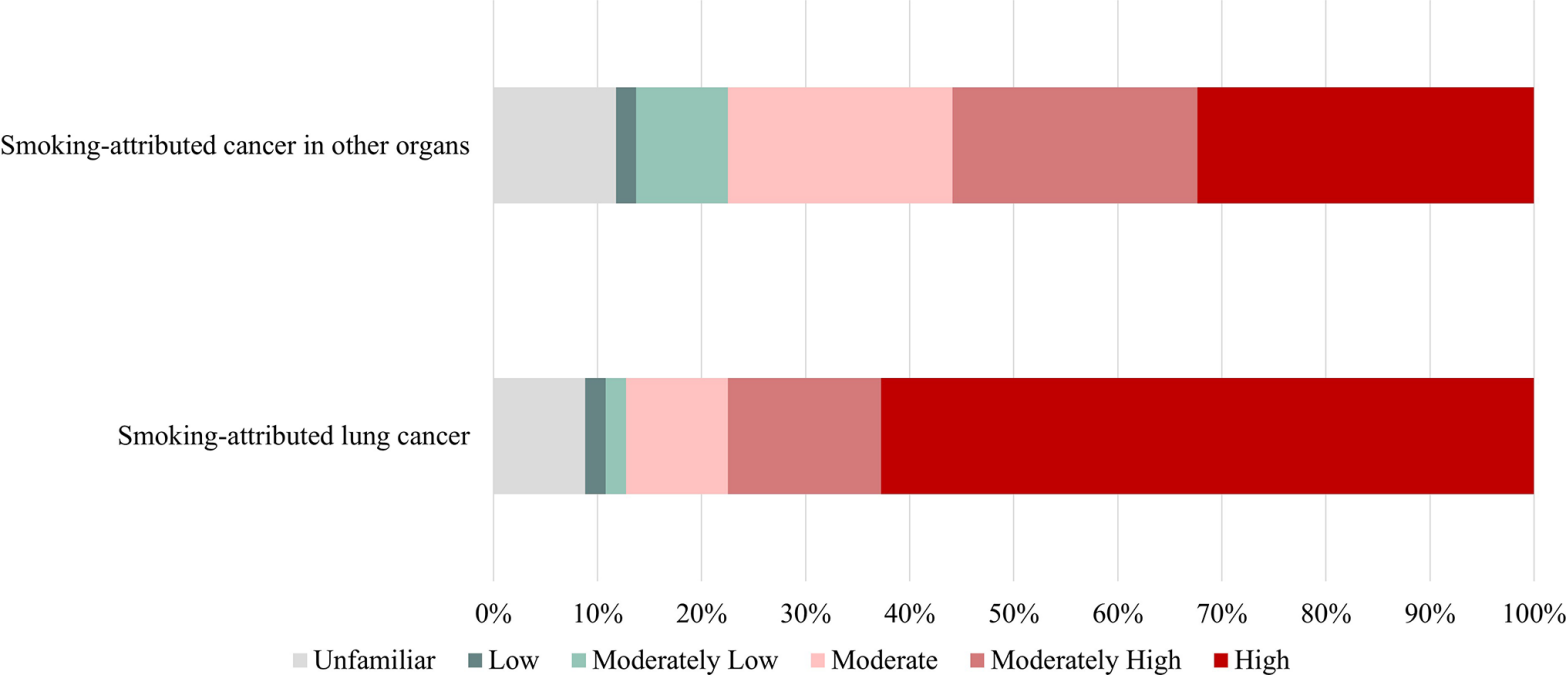
The impact of nicotine on the occurrence of diseases.

Participants were also asked to report their perception of nicotine’s impact on specific diseases. Results showed that 77.5% (high and moderately high) believed that nicotine played an active role in the development of smoking-related lung cancer. Additionally, approximately 55.9% of participants believed that nicotine had an active role in the development of cancers in organs outside of the lungs, such as the bladder, pancreas, and gastrointestinal tract (Figure 2). Furthermore, 28 believed that e-cigarettes were less addictive (compared to cigarettes).

In subgroup analyses, we observed noteworthy differences in knowledge and perceptions between smokers and non-smokers among the GPwSIs in Supplementary Table S1. Regarding the health risks associated with tobacco cigarettes, a higher percentage of smokers (57.7%) rated these risks as high compared to non-smokers (40.8%) (*p* = 0.20). Similarly, for e-cigarettes, while 17.1% of non-smokers considered them to have a high health risk, only 7.7% of smokers shared this view (*p* = 0.55). Interestingly, smokers demonstrated a better understanding of the correct dosage and usage of nicotine patches, with 50% answering correctly, compared to only 19.7% of non-smokers (*p* = 0.01). However, both groups showed similar levels of confidence in the safety of long-term (>6 months) NRT use, with no significant difference in their responses (*p* = 0.46). The analysis revealed that respondents’ age impacted their attitudes toward NRT. Those aged 20–39 were more likely to have a positive attitude toward the long-term use of NRT for smoking cessation compared to their older counterparts (*p* < 0.05) (Supplementary Tables S3).

## Discussion

This study aimed to assess the knowledge and awareness of GPwSIs in respiratory medicine regarding nicotine, NRT, and e-cigarettes. The study found that GPwSIs showed insufficient familiarity with the harms of smoking, NRT, oral smoking cessation medications, and e-cigarettes. This lack of familiarity was attributed to the possible absence of clinical guidelines for primary care and insufficient training on smoking cessation.

In this study, some GPwSIs were found to overestimate the risks of nicotine, despite its classification as a non-carcinogen (20). Misconceptions about the harmful effects of nicotine on human health are common among healthcare workers, which can be attributed to the belief that publicly downplaying the potential risks of nicotine may lead to an underestimation of smoking-related health problems (22). This viewpoint may create a bias against the use of NRT and oral smoking cessation medications, even though health organizations’ guidelines suggest that the risks of long-term nicotine use are far less than continued smoking (20). Consequently, there is a widespread stereotype of nicotine that leads some GPwSIs to discourage the long-term use of NRT for smokers.

Findings demonstrate that among GPwSIs, perceptions of harm and addictiveness of e-cigarettes is relatively diverse, possibly due to the inconsistent evaluation of e-cigarettes and the lack of unified guidelines (23). Current literature is insufficient to conclusively establish the effectiveness of e-cigarettes as a smoking cessation tool (24–26). However, a randomized controlled trial comparing e-cigarettes to NRT among adults who sought to quit smoking showed that the former had an advantage in achieving one-year abstinence rates (25). Although some health and medicine groups warn against e-cigarette use, others, including those in the United Kingdom and the United States, believe that e-cigarettes could be helpful in reducing the harm caused by smoking (12).

Smoking physicians are unlikely to advocate for smoking cessation to their patients (27). Over 25% of participants were current or former smokers, highlighting the persistent issue of smoking among GPwSIs. A meta-analysis on smoking rates among physicians revealed that family physicians and medical students had the highest proportion of smokers, and smoking rates among physicians in Europe and Asia were 40% higher than those in Oceania (27). Similarly, a meta-analysis on smoking among medical students in mainland China showed an overall smoking prevalence of 10.93% (range = 0.81 to 50.37%) in 65 studies involving 68,253 medical students, with a significantly higher prevalence among male medical students (28). As physicians serve as role models for patients and society, their smoking habits undermine their potential to persuade smoking patients to quit.

GPwSIs exhibited varying levels of knowledge and confidence in smoking cessation practices, including the use of NRT. This variation can be partly attributed to the diverse educational backgrounds of these practitioners. Typically, it appears that training in smoking cessation techniques and NRT is not consistently emphasized or standardized across programs (28). This education gap may contribute to the observed discrepancies in their approach to smoking cessation.

### Implementation

Assessing the knowledge of healthcare trainees holds key to customize educational plans based on actual training needs. Additionally, although GPwSIs are aware of their importance in the anti-tobacco movement, they lack optimal confidence in tobacco control strategies. Our study calls for revising the education combination for GPs in public health and preventive medicine.

### Limitations

The shortcomings of this study may have the following points. The first and foremost is that the study only included GPwSIs in respiratory medicine and may not accurately represent the views and knowledge of all Chinese GPs. Secondly, the study only focused on GPwSIs in respiratory medicine, who have relatively short practice experience in China and may have received different training and education, which could limit the generalizability of the findings. Thirdly, we did not explore the potential differences between GPwSIs in respiratory medicine and other GPs or respiratory specialists in terms of their knowledge and beliefs regarding smoking. More qualitative studies should be considered in future research. Nevertheless, our study succeeded in achieving its objectives by providing new insights into the perspectives of GPwSIs regarding e-cigarettes and NRT, and emphasizing the need for more research to enhance the knowledge of GPs in this field.

## Conclusion

The study highlights serious knowledge gaps among GPwSIs in respiratory medicine regarding nicotine, NRT, and e-cigarettes, which could hinder their ability to provide accurate and reliable advice to smokers. Therefore, it is imperative that GPwSIs receive appropriate training in tobacco cessation counseling to enable them to better assist tobacco users with quitting and integrate smoking cessation services into their routine care.

## Data availability statement

The raw data supporting the conclusions of this article will be made available by the authors, without undue reservation.

## Ethics statement

This study was approved by the Ethics Committee of West China Hospital, Sichuan University, Chengdu, China (No. 2021-1745). All the methods and protocols were performed in accordance with relevant guidelines and regulations and informed consent was obtained from all subjects. The studies were conducted in accordance with the local legislation and institutional requirements. The participants provided their informed consent to participate in this study.

## Author contributions

QZ and ZA conceived this study. QMZ, DY, ZW, and HZ designed the questionnaire. KA and LZ collected the data. QZ and HL analyzed the data. SL and CL illustrated the plots. QZ, KA, and ZA drafted the paper. All authors contributed to drafting or revising the article, have agreed on the journal to which the article will be submitted, gave final approval of the version to be published, and agreed to be accountable for all aspects of the work.

## Footnotes

^1^ https://www.wjx.cn/
