# Supplementary material for: Knowledge and awareness of nicotine, nicotine replacement therapy, and electronic cigarettes among general practitioners with a special interest in respiratory medicine in China
Source: Front Med (Lausanne). 2024 Jan 8;10:1236453. doi: 10.3389/fmed.2023.1236453 (PMC10805112; doi:10.3389/fmed.2023.1236453)
Supplement: Supplementary file 1 [file Table_1.docx]

**Supplementary information**

Table S1. Participants’ knowledge and awareness on nicotine, NRT, and electronic cigarettes. Differences between smokers and non-smokers.

| QUESTIONS | Non-smokers  No. (%) | Smokers  No. (%) | P |
| --- | --- | --- | --- |
| P2.1 Health risks score for tobacco cigarettes |  |  | 0.20 |
| High | 31(40.8) | 15(57.7) |  |
| Moderately High | 8(10.5) | 2(7.7) |  |
| Moderate | 10(13.2) | 5(19.2) |  |
| Moderately Low | 4(5.3) | 0(0.0) |  |
| Low | 3(3.9) | 2(7.7) |  |
| Unfamiliar | 20(26.3) | 2(7.7) |  |
| P2.2 Health risks score for E-cigarettes |  |  | 0.55 |
| High | 13(17.1) | 2(7.7) |  |
| Moderately High | 11(14.5) | 7(26.9) |  |
| Moderate | 13(17.1) | 4(15.4) |  |
| Moderately Low | 10(13.2) | 2(7.7) |  |
| Low | 9(11.8) | 5(19.2) |  |
| Unfamiliar | 20(26.3) | 6(23.1) |  |
| P2.3 Health risks score for NRT |  |  | 0.28 |
| High | 1(1.3) | 0(0.0) |  |
| Moderately High | 3(3.9) | 4(15.4) |  |
| Moderate | 17(22.4) | 7(26.9) |  |
| Moderately Low | 12(15.8) | 3(11.5) |  |
| Low | 13(17.1) | 6(23.1) |  |
| Unfamiliar | 30(39.5) | 6(23.1) |  |
| P2.4 Health risks score for oral smoking cessation medications |  |  | 0.21 |
| High | 2(2.6) | 1(3.8) |  |
| Moderately High | 4(5.3) | 2(7.7) |  |
| Moderate | 17(22.4) | 8(30.8) |  |
| Moderately Low | 12(15.8) | 6(23.1) |  |
| Low | 10(13.2) | 5(19.2) |  |
| Unfamiliar | 31(40.8) | 4(15.4) |  |
| P2.5 Approved smoking cessation medications in China |  |  | 1.00 |
| Right | 38(50.0) | 13(50.0) |  |
| Wrong | 38(50.0) | 13(50.0) |  |
| P2.6 Which of the following is correct regarding the dosage of nicotine patches? |  |  | 0.01 |
| Right | 15(19.7) | 13(50.0) |  |
| Wrong | 13(17.1) | 2(7.7) |  |
| Unfamiliar | 48(63.2) | 11(42.3) |  |
| P2.7 Do you believe that long-term (>6 months) use of NRT can reduce smoking or help quit smoking, and is relatively safe? |  |  | 0.46 |
| Not safe | 26(34.2) | 11(42.3) |  |
| Safe | 50(65.8) | 15(57.7) |  |
| P2.8 Do you recommend long-term (>6 months) use of NRT to treat those who cannot reduce or quit smoking in the short term? |  |  | 0.30 |
| Not recommended | 21(27.6) | 10(38.5) |  |
| Recommended | 55(72.4) | 16(61.5) |  |
| P2.9 Do you think e-cigarettes are a tool for quitting smoking? |  |  | 0.50 |
| Yes | 21(27.6) | 9(34.6) |  |
| No | 55(72.4) | 17(65.4) |  |
| P2.10 Can e-cigarettes be addictive? |  |  | 0.11 |
| Yes | 44(57.9) | 14(53.8) |  |
| No | 4(5.3) | 5(19.2) |  |
| Unfamiliar | 28(36.8) | 7(26.9) |  |
| P2.11 Would you recommend e-cigarettes as a substitute for cigarettes to smokers? |  |  | 0.42 |
| Yes | 20(26.3) | 9(34.6) |  |
| No | 56(73.7) | 17(65.4) |  |
| P2.12 The Impact of Nicotine on Smoking-Related Lung Cancer |  |  | 0.61 |
| High | 47(61.8) | 17(65.4) |  |
| Moderately High | 12(15.8) | 3(11.5) |  |
| Moderate | 6(7.9) | 4(15.4) |  |
| Moderately Low | 2(2.6) | 0(0.0) |  |
| Low | 1(1.3) | 1(3.8) |  |
| Unfamiliar | 8(10.5) | 1(3.8) |  |
| P2.13 The impact of nicotine on smoking-related tumors in other organs |  |  | 0.39 |
| High | 25(32.9) | 8(30.8) |  |
| Moderately High | 17(22.4) | 7(26.9) |  |
| Moderate | 14(18.4) | 8(30.8) |  |
| Moderately Low | 8(10.5) | 1(3.8) |  |
| Low | 1(1.3) | 1(3.8) |  |
| Unfamiliar | 11(14.5) | 1(3.8) |  |
| P2.14 The addictiveness of NRT compared to cigarettes |  |  | 0.01 |
| Higher | 6(7.9) | 4(15.4) |  |
| Equal | 1(1.3) | 5(19.2) |  |
| Lower | 33(43.4) | 10(38.5) |  |
| Unfamiliar | 36(47.4) | 7(26.9) |  |
| P2.15 The addictiveness of E-cigarettes compared to cigarettes |  |  | 0.52 |
| Higher | 7(9.2) | 4(15.4) |  |
| Equal | 14(18.4) | 4(15.4) |  |
| Lower | 19(25.0) | 9(34.6) |  |
| Unfamiliar | 36(47.4) | 9(34.6) |  |

*Notes*: E-cigarettes: Electronic cigarettes; NRT: Nicotine replacement therapy

Table S2. Participants’ knowledge and awareness on nicotine, NRT, and electronic cigarettes. Differences between age groups.

| QUESTIONS | Age 20-39  No. (%) | Age ≥ 40  No. (%) | P |
| --- | --- | --- | --- |
| P2.1 Health risks score for tobacco cigarettes |  |  | 0.04 |
| High | 22(62.9) | 24(35.8) |  |
| Moderately High | 1(2.9) | 9(13.4) |  |
| Moderate | 4(11.4) | 11(16.4) |  |
| Moderately Low | 0(0.0) | 4(6.0) |  |
| Low | 3(8.6) | 2(3.0) |  |
| Unfamiliar | 5(14.3) | 17(25.4) |  |
| P2.2 Health risks score for E-cigarettes |  |  | 0.18 |
| High | 5(14.3) | 10(14.9) |  |
| Moderately High | 7(20.0) | 11(16.4) |  |
| Moderate | 10(28.6) | 7(10.4) |  |
| Moderately Low | 3(8.6) | 9(13.4) |  |
| Low | 2(5.7) | 12(17.9) |  |
| Unfamiliar | 8(22.9) | 18(26.9) |  |
| P2.3 Health risks score for NRT |  |  | 0.27 |
| High | 1(2.9) | 0(0.0) |  |
| Moderately High | 1(2.9) | 6(9.0) |  |
| Moderate | 11(31.4) | 13(19.4) |  |
| Moderately Low | 5(14.3) | 10(14.9) |  |
| Low | 8(22.9) | 11(16.4) |  |
| Unfamiliar | 9(25.7) | 27(40.3) |  |
| P2.4 Health risks score for oral smoking cessation medications |  |  | 0.27 |
| High | 2(5.7) | 1(1.5) |  |
| Moderately High | 0(0.0) | 6(9.0) |  |
| Moderate | 10(28.6) | 15(22.4) |  |
| Moderately Low | 8(22.9) | 10(14.9) |  |
| Low | 5(14.3) | 10(14.9) |  |
| Unfamiliar | 10(28.6) | 25(37.3) |  |
| P2.5 Approved smoking cessation medications in China | ○ |  | 1.00 |
| Right | 17(48.6) | 34(50.7) |  |
| Wrong | 18(51.4) | 33(49.3) |  |
| P2.6 Which of the following is correct regarding the dosage of nicotine patches? |  |  | 0.59 |
| Right | 8(22.9) | 20(29.9) |  |
| Wrong | 4(11.4) | 11(16.4) |  |
| Unfamiliar | 23(65.7) | 36(53.7) |  |
| P2.7 Do you believe that long-term (>6 months) use of NRT can reduce smoking or help quit smoking, and is relatively safe? |  |  | 0.52 |
| Not safe | 11(31.4) | 26(38.8) |  |
| Safe | 24(68.6) | 41(61.2) |  |
| P2.8 Do you recommend long-term (>6 months) use of NRT to treat those who cannot reduce or quit smoking in the short term? |  |  | 0.01 |
| Not recommended | 5(14.3) | 26(38.8) |  |
| Recommended | 30(85.7) | 41(61.2) |  |
| P2.9 Do you think e-cigarettes are a tool for quitting smoking? |  |  | 0.65 |
| Yes | 9(25.7) | 21(31.3) |  |
| No | 26(74.3) | 46(68.7) |  |
| P2.10 Can e-cigarettes be addictive? |  |  | 0.63 |
| Yes | 22(62.9) | 36(53.7) |  |
| No | 2(5.7) | 7(10.4) |  |
| Unfamiliar | 11(31.4) | 24(35.8) |  |
| P2.11 Would you recommend e-cigarettes as a substitute for cigarettes to smokers? |  |  | 0.63 |
| Yes | 11(31.4) | 18(26.9) |  |
| No | 24(68.6) | 49(73.1) |  |
| P2.12 The Impact of Nicotine on Smoking-Related Lung Cancer |  |  | 0.69 |
| High | 22(62.9) | 42(62.7) |  |
| Moderately High | 3(8.6) | 12(17.9) |  |
| Moderate | 4(11.4) | 6(9.0) |  |
| Moderately Low | 1(2.9) | 1(1.5) |  |
| Low | 1(2.9) | 1(1.5) |  |
| Unfamiliar | 4(11.4) | 5(7.5) |  |
| P2.13 The impact of nicotine on smoking-related tumors in other organs |  |  | 0.21 |
| High | 12(34.3) | 21(31.3) |  |
| Moderately High | 7(20.0) | 17(25.4) |  |
| Moderate | 7(20.0) | 15(22.4) |  |
| Moderately Low | 1(2.9) | 8(11.9) |  |
| Low | 2(5.7) | 0(0.0) |  |
| Unfamiliar | 6(17.1) | 6(9.0) |  |
| P2.14 The addictiveness of NRT compared to cigarettes |  |  | 0.87 |
| Higher | 3(8.6) | 7(10.4) |  |
| Equal | 3(8.6) | 3(4.5) |  |
| Lower | 15(42.9) | 28(41.8) |  |
| Unfamiliar | 14(40.0) | 29(43.3) |  |
| P2.15 The addictiveness of E-cigarettes compared to cigarettes |  |  | 0.66 |
| Higher | 4(11.4) | 7(10.4) |  |
| Equal | 7(20.0) | 11(16.4) |  |
| Lower | 7(20.0) | 21(31.3) |  |
| Unfamiliar | 17(48.6) | 28(41.8) |  |

*Notes*: E-cigarettes: Electronic cigarettes; NRT: Nicotine replacement therapy

Table S3. Participants’ knowledge and awareness on nicotine, NRT, and electronic cigarettes. Differences between gender groups.

| QUESTIONS | Male  No. (%) | Female  No. (%) | P |
| --- | --- | --- | --- |
| P2.1 Health risks score for tobacco cigarettes |  |  | 0.33 |
| High | 24(46.2) | 22(44.0) |  |
| Moderately High | 5(9.6) | 5(10.0) |  |
| Moderate | 10(19.2) | 5(10.0) |  |
| Moderately Low | 3(5.8) | 1(2.0) |  |
| Low | 3(5.8) | 2(4.0) |  |
| Unfamiliar | 7(13.5) | 15(30.0) |  |
| P2.2 Health risks score for E-cigarettes |  |  | 0.21 |
| High | 5(9.6) | 10(20.0) |  |
| Moderately High | 9(17.3) | 9(18.0) |  |
| Moderate | 13(25.0) | 4(8.0) |  |
| Moderately Low | 5(9.6) | 7(14.0) |  |
| Low | 8(15.4) | 6(12.0) |  |
| Unfamiliar | 12(23.1) | 14(28.0) |  |
| P2.3 Health risks score for NRT |  |  | 0.04 |
| High | 1(1.9) | 0(0.0) |  |
| Moderately High | 3(5.8) | 4(8.0) |  |
| Moderate | 16(30.8) | 8(16.0) |  |
| Moderately Low | 9(17.3) | 6(12.0) |  |
| Low | 12(23.1) | 7(14.0) |  |
| Unfamiliar | 11(21.2) | 25(50.0) |  |
| P2.4 Health risks score for oral smoking cessation medications |  |  | 0.01 |
| High | 2(3.8) | 1(2.0) |  |
| Moderately High | 2(3.8) | 4(8.0) |  |
| Moderate | 17(32.7) | 8(16.0) |  |
| Moderately Low | 11(21.2) | 7(14.0) |  |
| Low | 10(19.2) | 5(10.0) |  |
| Unfamiliar | 10(19.2) | 25(50.0) |  |
| P2.5 Approved smoking cessation medications in China | ○ |  | 1.00 |
| Right | 26(50.0) | 25(50.0) |  |
| Wrong | 26(50.0) | 25(50.0) |  |
| P2.6 Which of the following is correct regarding the dosage of nicotine patches? |  |  | 0.05 |
| Right | 18(34.6) | 10(20.0) |  |
| Wrong | 10(19.2) | 5(10.0) |  |
| Unfamiliar | 24(46.2) | 35(70.0) |  |
| P2.7 Do you believe that long-term (>6 months) use of NRT can reduce smoking or help quit smoking, and is relatively safe? |  |  | 0.64 |
| Not safe | 20(38.5) | 17(34.0) |  |
| Safe | 32(61.5) | 33(66.0) |  |
| P2.8 Do you recommend long-term (>6 months) use of NRT to treat those who cannot reduce or quit smoking in the short term? |  |  | 0.73 |
| Not recommended | 15(28.8) | 16(32.0) |  |
| Recommended | 37(71.2) | 34(68.0) |  |
| P2.9 Do you think e-cigarettes are a tool for quitting smoking? |  |  | 0.11 |
| Yes | 19(36.5) | 11(22.0) |  |
| No | 33(63.5) | 39(78.0) |  |
| P2.10 Can e-cigarettes be addictive? |  |  | 0.12 |
| Yes | 31(59.6) | 27(54.0) |  |
| No | 7(13.5) | 2(4.0) |  |
| Unfamiliar | 14(26.9) | 21(42.0) |  |
| P2.11 Would you recommend e-cigarettes as a substitute for cigarettes to smokers? |  |  | 0.06 |
| Yes | 19(36.5) | 10(20.0) |  |
| No | 33(63.5) | 40(80.0) |  |
| P2.12 The Impact of Nicotine on Smoking-Related Lung Cancer |  |  | 0.69 |
| High | 29(55.8) | 35(70.0) |  |
| Moderately High | 9(17.3) | 6(12.0) |  |
| Moderate | 6(11.5) | 4(8.0) |  |
| Moderately Low | 1(1.9) | 1(2.0) |  |
| Low | 2(3.8) | 0(0.0) |  |
| Unfamiliar | 5(9.6) | 4(8.0) |  |
| P2.13 The impact of nicotine on smoking-related tumors in other organs |  |  | 0.55 |
| High | 15(28.8) | 18(36.0) |  |
| Moderately High | 13(25.0) | 11(22.0) |  |
| Moderate | 9(17.3) | 13(26.0) |  |
| Moderately Low | 6(11.5) | 3(6.0) |  |
| Low | 2(3.8) | 0(0.0) |  |
| Unfamiliar | 7(13.5) | 5(10.0) |  |
| P2.14 The addictiveness of NRT compared to cigarettes |  |  | 0.21 |
| Higher | 7(13.5) | 3(6.0) |  |
| Equal | 5(9.6) | 1(2.0) |  |
| Lower | 20(38.5) | 23(46.0) |  |
| Unfamiliar | 20(38.5) | 23(46.0) |  |
| P2.15 The addictiveness of E-cigarettes compared to cigarettes |  |  | 0.53 |
| Higher | 5(9.6) | 6(12.0) |  |
| Equal | 7(13.5) | 11(22.0) |  |
| Lower | 17(32.7) | 11(22.0) |  |
| Unfamiliar | 23(44.2) | 22(44.0) |  |

*Notes*: E-cigarettes: Electronic cigarettes; NRT: Nicotine replacement therapy
