# Supplementary material for: Knowledge and awareness of nicotine, nicotine replacement therapy, and electronic cigarettes among general practitioners with a special interest in respiratory medicine in China
Source: Front Med (Lausanne). 2024 Jan 8;10:1236453. doi: 10.3389/fmed.2023.1236453 (PMC10805112; doi:10.3389/fmed.2023.1236453)
Supplement: Supplementary file 2 [file Data_Sheet_1.docx]

**Survey on general practitioners' knowledge and attitudes towards nicotine, nicotine replacement therapy, and electronic cigarettes**

This survey is anonymous and the purpose is only to understand general practitioners' knowledge and attitudes towards nicotine, nicotine replacement therapy, and electronic cigarettes.

It is not an exam or a test. Please cooperate and submit your responses. Thank you.

The following questions are multiple-choice.

**Part 1: Background Information**

P1.1 Gender:

○ Male

○ Female

P1.2 Age:

○ 20-29

○ 30-39

○ 40-49

○ 50-

P1.3 Professional title:

○ Primary

○ Intermediate

○ Senior

P1.4 Highest academic degree obtained:

○ Associate degree

○ Bachelor degree

○ Master or above degree

P1.5 Hospital type:

○ Tertiary hospital

○ Secondary hospital

○ Primary hospital

P1.6 Your smoking status?

○ Never smoked

○ Quit smoking

○ Still smoking

P1.7 Have you tried to help patients quit smoking? Which method(s) have you used? [Multiple-choice] *

□ Through the use of oral smoking cessation medications

□ Through the use of nicotine replacement therapy (patches, gum, inhalers, nasal sprays, etc.)

□ Through the use of systematic psychological support

□ Using other methods

□ Have not attempted to help patients quit smoking

P1.8 In your daily consultations, do you actively ask and record the smoking history of the patient? [Single-choice] *

○ Never

○ Occasionally

○ Sometimes

○ Often

○ Always

**Part 2: Knowledge and Awareness Assessment**

Based on your knowledge, please rate the health risks of the following items [Matrix scale question] *

Nicotine replacement therapy drugs: such as nicotine patches and nicotine gum.

|  | Unfamiliar | Low | Moderately Low | Moderate | Moderately High | High |
| --- | --- | --- | --- | --- | --- | --- |
| P2.1 Tobacco cigarettes | ○ | ○ | ○ | ○ | ○ | ○ |
| P2.2 E-cigarettes | ○ | ○ | ○ | ○ | ○ | ○ |
| P2.3 Nicotine replacement therapy | ○ | ○ | ○ | ○ | ○ | ○ |
| P2.4 Oral smoking cessation medications (Varenicline, Bupropion) | ○ | ○ | ○ | ○ | ○ | ○ |

P2.5 Currently approved smoking cessation medications in China include: [Multiple-choice]

○ Nicotine patch

○ Nicotine gum

○ Bupropion hydrochloride sustained-release tablets

○ Varenicline

P2.6 Which of the following is correct regarding the dosage of nicotine patches? [Single choice question] *

○ Start with a higher dose and gradually reduce according to the treatment course

○ Start with a lower dose and gradually increase according to the treatment course

○ Fixed dose and treatment course

○ Unfamiliar/Lack of knowledge

P2.7 Do you believe that long-term (>6 months) use of nicotine replacement therapy can reduce smoking or help quit smoking, and is relatively safe? [Single choice question] *

○ Not safe

○ Safe

P2.8 Do you recommend long-term (>6 months) use of nicotine replacement therapy to treat those who cannot reduce or quit smoking in the short term? [Single choice question]

○ Not recommended

○ Recommended

P2.9 Do you think e-cigarettes are a tool for quitting smoking? [Single choice question]

○ Yes, a tool for quitting smoking

○ No, not a tool for quitting smoking

P2.10 Can e-cigarettes be addictive? [Single choice question] *

○ Addictive

○ Not addictive

○ Don't know

P2.11 Would you recommend e-cigarettes as a substitute for cigarettes to smokers? [Single choice question] *

○ No, I would not recommend it

○ Yes, I would recommend it

Rate the impact of nicotine on the following diseases [Matrix scale question] *

|  | Unfamiliar | Low | Moderately Low | Moderate | Moderately High | High |
| --- | --- | --- | --- | --- | --- | --- |
| P2.12 Smoking-related lung cancer | ○ | ○ | ○ | ○ | ○ | ○ |
| P2.13 Smoking-related other organ tumors (such as bladder, pancreas, gastrointestinal tract, etc.) | ○ | ○ | ○ | ○ | ○ | ○ |

Compared to cigarettes, [Matrix single choice question] *

|  | Higher | Equal | Lower | Unfamiliar |
| --- | --- | --- | --- | --- |
| P2.14 The addictiveness of nicotine replacement products | ○ | ○ | ○ | ○ |
| P2.15 The addictiveness of e-cigarettes | ○ | ○ | ○ | ○ |

P2.16 Rate your confidence level in your ability to provide smoking cessation services [Single choice question] *

○ Not at all confident

○ Somewhat not confident

○ Somewhat confident

○ Quite confident

○ Very confident

P2.17 Do you need training on smoking cessation? [Single-choice] *

○ Yes

○ No
